# Supplementary material for: Italian adaptation of the Multidimensional Iowa Suggestibility Scale (MISS)
Source: PeerJ. 2024 Apr 29;12:e17145. doi: 10.7717/peerj.17145 (PMC11064866; doi:10.7717/peerj.17145)
Supplement: Supplemental Information 1 — The file contains both the Study 1 and the study 2 versions in the adapted language (Italian) and in the original language (English) [file peerj-12-17145-s001.docx]

**Study 1.**

*Italian version*

- Multidimensional Iowa Suggestibility Scale (Kotov, Bellman & Watson, 2004).

Per favore, esprima il grado di accordo rispetto alle seguenti affermazioni:

Per niente d’accordo; Poco d’accordo; Moderatamente d’accordo; Abbastanza d’accordo; Molto d’accordo.

1. Gli spot pubblicitari mi fanno venire voglia di prodotti che non pensavo mi servissero
2. Ottengo informazioni dagli spot pubblicitari
3. Posso essere influenzato da una efficace pubblicità
4. Dopo che qualcuno che conosco prova un nuovo prodotto, voglio provarlo anche io
5. Voglio un prodotto perché mi piace che la persona che lo promuove
6. Quando un addetto alle vendite mostra i vantaggi del suo servizio, riesce a convincermi
7. Un buon venditore può davvero farmi venire voglia del suo prodotto
8. Ottengo buoni consigli per gli acquisti dai giornali o dalla tv
9. Se un prodotto è messo ben in vista, di solito lo voglio comprare
10. Seguo lo stile dei personaggi famosi
11. Seguo gli spot pubblicitari come guida per fare shopping
12. Un ragionamento logico può farmi cambiare idea
13. Posso essere convinta/o da un buon ragionamento
14. Trovo che il consiglio delle persone può essere utile nel prendere delle decisioni
15. Nel prendere una decisione, seguo i consigli degli altri
16. Non mi dispiace cambiare idea, dopo aver ascoltato differenti punti di vista
17. Più sono esposto al punto di vista di altre persone, più il mio punto di vista cambia
18. Mi vengono molte buone idee dagli altri
19. Ho fiducia nei consigli degli esperti
20. Se avessi una opinione che gli altri non condividono, potrei seriamente metterla in discussione
21. Riesco ad essere convinto/a da un articolo di giornale ben scritto.
22. Sono facilmente influenzato dalla opinione di altre persone
23. In una discussione, uso delle argomentazioni che ho ascoltato da altre persone
24. Quando si parla di politica, mi trovo ad usare argomentazioni che ho recentemente letto o ascoltato in tv
25. Mi capita di cambiare la mia opinione dopo aver parlato con altri
26. Dopo aver visto la pubblicità di un deodorante, mi accorgo di avere un cattivo odore
27. Se mi si dice che non ho un bell’aspetto, comincio a stare male
28. Non mi accorgo di essere stanco, fino a quando qualcuno non me lo dice direttamente
29. Quando qualcuno si schiarisce la voce, mi accorgo di sentire la gola irritata
30. Dopo aver sentito parlare di una malattia, a volte comincio a sentire i sintomi di quella malattia
31. Quando qualcuno tossisce o starnutisce, sento il bisogno di fare la stessa cosa
32. Quando vedo qualcuno avere i brividi di freddo, vengono anche a me
33. Quando la gente mi dice come si sente, mi accorgo di sentirmi allo stesso modo
34. Quando qualcuno descrive una esperienza, a volte mi sento come se la stessi vivendo anche io
35. Non mi rendo conto che un ambiente è troppo caldo fino a quando non lo dice qualcun altro
36. Essere in una stanza in cui qualcuno sta dormendo, mi rende assonnato
37. Dopo aver visto la pubblicità di una crema, sento la mia pelle secca
38. Dopo aver visto un film di paura, mi sento teso per un po’
39. Una scena toccante mi fa venire gli occhi lucidi
40. Pensare a qualcosa di spaventoso, mi fa venire le palpitazioni
41. Dopo aver visto qualcosa di impressionante, questa immagine mi torna in mente
42. Quando qualcuno sbadiglia, sbadiglio pure io
43. Mi sento più attraente se qualcuno mi fa dei complimenti per il mio aspetto
44. Leggere la descrizione di piatti gustosi mi fa venire l’acquolina in bocca
45. Immaginare una bevanda dissetante, mi fa venire sete
46. L’odore del cibo, di solito, mi fa venire fame
47. Quando ascolto della musica, il mio stato d’animo cambia di conseguenza
48. Quando penso a qualcosa di piacevole, mi accorgo di stare sorridendo
49. Quando leggo una storia, sento quello che sta vivendo il personaggio
50. Un buon film mi coinvolge emotivamente
51. A me e ai miei amici, piacciono le stesse cose
52. A me e ai miei amici piacciono gli stessi negozi
53. Mi piace il modo in cui vestono i miei amici
54. Mi vesto in modo molto diverso dai miei amici
55. Non mi piacciono i film che piacciono ai miei amici
56. Mi sembra di avere una prospettiva sulla vita che è molto simile a quella delle persone che mi circondano
57. Compro le stesse cose che hanno i miei amici
58. Condivido le opinioni dei miei amici
59. Io e i miei amici abbiamo gli stessi gusti musicali
60. Mi piacciono le stesse celebrità dei miei amici
61. Le mie cose preferite sono le stesse dei miei amici
62. Seguo la moda del momento
63. Mi devo adeguare agli altri
64. Seguo le abitudini dei miei amici

*English version*

- Please express the degree of agreement with respect to the following statements:

Not at all agree; Slightly agree; Moderately agree; Somewhat agree; Very agree.

1. Commercials sometimes make me want products that I did not know I needed
2. I often get information about products from commercials
3. I can be influenced by a good commercial
4. After someone I know tries a new product, I will usually try it too
5. Sometimes I want a product because I like the person endorsing it
6. When a salesperson explains advantages of their service, I am usually pretty convinced
7. A good salesperson can really make me want their product
8. I get a lot of good practical advice from magazines or TV
9. If a product is nicely displayed, I usually want to buy it
10. I get my style from certain celebrities
11. I use advertisements as a guide for shopping
12. A logical argument can make me change my mind
13. I can be convinced by a good argument
14. I find other people’s advice helpful in making decisions
15. When making a decision, I often follow other people’s advice
16. I don’t mind changing my opinion after hearing a different point of view
17. The more I am exposed to other people’s views, the more my own view of the world changes
18. I get many good ideas from others
19. I trust the advice of experts
20. If I had an opinion that no one else shared, I would seriously question it
21. I usually can be persuaded by a well-written editorial
22. I am easily influenced by other people’s opinions
23. In a discussion I often use arguments that I’ve heard other people make
24. When discussing politics I often find myself using arguments that I recently read or heard on TV
25. I frequently change my opinion after talking with others
26. After watching deodorant commercials, I sometimes notice that I smell
27. If I am told I don’t look well, I start feeling ill
28. I sometimes don’t realize that I am tired until someone tells me I look tired
29. When someone clears their throat, I often notice that my throat feels scratchy
30. After hearing about an illness, I sometimes start feeling symptoms of that illness
31. When someone coughs or sneezes, I usually feel the urge to do the same
32. When I see someone shiver, I often feel a chill myself
33. When people tell me how they feel, I often notice that I feel the same way
34. When someone describes an experience, I sometimes feel as if I am having it
35. I sometimes don’t realize that a room is too hot until someone else mentions it
36. Being in a room where someone is sleeping makes me sleepy
37. After I see a commercial for lotion, sometimes my skin feels dry
38. After seeing a scary movie I feel jumpy for a while
39. A touching scene can make my eyes water
40. Thinking about something scary can make my heart pound
41. After seeing something striking, the image often comes back to me
42. When someone yawns, I usually yawn myself
43. I feel more attractive if someone compliments me on my appearance
44. Reading descriptions of tasty dishes can make my mouth water
45. Imagining a refreshing drink can make me thirsty
46. The smell of food usually makes me hungry
47. When I listen to music my mood usually changes accordingly
48. When I think about something pleasant I sometimes notice that I am smiling
49. When I read a story I sometimes feel what the character goes through
50. I often get emotionally involved in a good movie
51. My friends and I like all the same things
52. My friends and I like the same stores
53. I like the style of clothes that my friends wear
54. I dress very differently from my friends
55. I don’t like most of the movies my friends like
56. I seem to have a perspective on life that is quite similar to the people around me
57. I often buy things that my friends have
58. I share many of my friends’ opinions
59. My friends and I have similar music tastes
60. I like the same celebrities as my friends
61. I discovered many of my favorite things through my friends
62. I follow current fashion trends
63. It is important for me to fit in
64. I have picked-up many habits from my friends

**Study 2.**

*Italian version*

- Per favore, esprima il grado di accordo rispetto alle seguenti affermazioni:
- Per niente d’accordo; Poco d’accordo; Moderatamente d’accordo; Abbastanza d’accordo; Molto d’accordo.

1. Gli spot pubblicitari mi fanno venire voglia di prodotti che non pensavo mi servissero
2. Ottengo informazioni dagli spot pubblicitari
3. Posso essere influenzato da una efficace pubblicità
4. Dopo che qualcuno che conosco prova un nuovo prodotto, voglio provarlo anche io
5. Quando un addetto alle vendite mostra i vantaggi del suo servizio, riesce a convincermi
6. Un buon venditore può davvero farmi venire voglia del suo prodotto
7. Ottengo buoni consigli per gli acquisti dai giornali o dalla tv
8. Se un prodotto è messo ben in vista, di solito lo voglio comprare
9. Posso essere convinta/o da un buon ragionamento
10. Trovo che il consiglio delle persone può essere utile nel prendere delle decisioni
11. Nel prendere una decisione, seguo i consigli degli altri
12. Non mi dispiace cambiare idea, dopo aver ascoltato differenti punti di vista
13. Più sono esposto al punto di vista di altre persone, più il mio punto di vista cambia
14. Mi vengono molte buone idee dagli altri
15. Ho fiducia nei consigli degli esperti
16. Se avessi una opinione che gli altri non condividono, potrei seriamente metterla in discussione
17. Riesco ad essere convinto/a da un articolo di giornale ben scritto.
18. Sono facilmente influenzato dalla opinione di altre persone
19. In una discussione, uso delle argomentazioni che ho ascoltato da altre persone
20. Quando si parla di politica, mi trovo ad usare argomentazioni che ho recentemente letto o ascoltato in tv
21. Mi capita di cambiare la mia opinione dopo aver parlato con altri
22. Dopo aver visto un film di paura, mi sento teso per un po’
23. Una scena toccante mi fa venire gli occhi lucidi
24. Pensare a qualcosa di spaventoso, mi fa venire le palpitazioni
25. Dopo aver visto qualcosa di impressionante, questa immagine mi torna in mente
26. Quando qualcuno sbadiglia, sbadiglio pure io
27. Mi sento più attraente se qualcuno mi fa dei complimenti per il mio aspetto
28. Leggere la descrizione di piatti gustosi mi fa venire l’acquolina in bocca
29. Immaginare una bevanda dissetante, mi fa venire sete
30. L’odore del cibo, di solito, mi fa venire fame
31. Quando ascolto della musica, il mio stato d’animo cambia di conseguenza
32. Quando penso a qualcosa di piacevole, mi accorgo di stare sorridendo
33. Quando leggo una storia, sento quello che sta vivendo il personaggio
34. Un buon film mi coinvolge emotivamente
35. A me e ai miei amici, piacciono le stesse cose
36. A me e ai miei amici piacciono gli stessi negozi
37. Mi piace il modo in cui vestono i miei amici
38. Mi sembra di avere una prospettiva sulla vita che è molto simile a quella delle persone che mi circondano
39. Compro le stesse cose che hanno i miei amici
40. Condivido le opinioni dei miei amici
41. Io e i miei amici abbiamo gli stessi gusti musicali
42. Mi piacciono le stesse celebrità dei miei amici
43. Le mie cose preferite sono le stesse dei miei amici
44. Seguo la moda del momento
45. Mi devo adeguare agli altri
46. Seguo le abitudini dei miei amici

*English version*

- Please express the degree of agreement with respect to the following statements:

Not at all agree; Slightly agree; Moderately agree; Somewhat agree; Very agree.

1. Commercials sometimes make me want products that I did not know I needed
2. I often get information about products from commercials
3. I can be influenced by a good commercial
4. After someone I know tries a new product, I will usually try it too
5. When a salesperson explains advantages of their service, I am usually pretty convinced
6. A good salesperson can really make me want their product
7. I get a lot of good practical advice from magazines or TV
8. If a product is nicely displayed, I usually want to buy it
9. I can be convinced by a good argument
10. I find other people’s advice helpful in making decisions
11. When making a decision, I often follow other people’s advice
12. I don’t mind changing my opinion after hearing a different point of view
13. The more I am exposed to other people’s views, the more my own view of the world changes
14. I get many good ideas from others
15. I trust the advice of experts
16. If I had an opinion that no one else shared, I would seriously question it
17. I usually can be persuaded by a well-written editorial
18. I am easily influenced by other people’s opinions
19. In a discussion I often use arguments that I’ve heard other people make
20. When discussing politics I often find myself using arguments that I recently read or heard on TV
21. I frequently change my opinion after talking with others
22. After seeing a scary movie I feel jumpy for a while
23. A touching scene can make my eyes water
24. Thinking about something scary can make my heart pound
25. After seeing something striking, the image often comes back to me
26. When someone yawns, I usually yawn myself
27. I feel more attractive if someone compliments me on my appearance
28. Reading descriptions of tasty dishes can make my mouth water
29. Imagining a refreshing drink can make me thirsty
30. The smell of food usually makes me hungry
31. When I listen to music my mood usually changes accordingly
32. When I think about something pleasant I sometimes notice that I am smiling
33. When I read a story I sometimes feel what the character goes through
34. I often get emotionally involved in a good movie
35. My friends and I like all the same things
36. My friends and I like the same stores
37. I like the style of clothes that my friends wear
38. I seem to have a perspective on life that is quite similar to the people around me
39. I often buy things that my friends have
40. I share many of my friends’ opinions
41. My friends and I have similar music tastes
42. I like the same celebrities as my friends
43. I discovered many of my favorite things through my friends
44. I follow current fashion trends
45. It is important for me to fit in
46. I have picked-up many habits from my friends
